# Improved global protein homolog detection with major gains in function identification

**DOI:** 10.1073/pnas.2211823120

**Published:** 2023-02-24

**Authors:** Mesih Kilinc, Kejue Jia, Robert L. Jernigan

**Affiliations:** ^a^Bioinformatics and Computational Biology Program, Iowa State University, Ames, IA 50011; ^b^Roy J. Carver Department of Biochemistry, Biophysics and Molecular Biology, Iowa State University, Ames, IA 50011

**Keywords:** sequence search, homolog, proteins, function identification, protein language models

## Abstract

There are several hundred million protein sequences, but the relationships among them are not fully available from existing homolog detection methods. There is an essential need for an improved method to push homolog detection to lower levels of sequence identity. The method used here relies on a language model to represent proteins numerically in a matrix (an embedding) and uses discrete cosine transforms to compress the data to extract the most essential part, significantly reducing the data size. This PRotein Ortholog Search Tool (PROST) is significantly faster with linear runtimes, and most importantly, computes the distances between pairs of protein sequences to yield homologs at significantly lower levels of sequence identity than previously. The extent of allosteric effects in proteins points out the importance of global aspects of structure and sequence. PROST excels at global homology detection but not at detecting local homologs. Results are validated by strong similarities between the corresponding pairs of structures. The number of remote homologs detected increased significantly and pushes the effective sequence matches more deeply into the twilight zone. Human protein sequences presently having no assigned function now find significant numbers of putative homologs for 93% of cases and structurally verified assigned functions for 76.4% of these cases. The data compression enables massive searches for homologs with short search times while yielding significant gains in the numbers of remote homologs detected. The method is sufficiently efficient to permit whole-genome/proteome comparisons. The PROST web server is accessible at https://mesihk.github.io/prost.

## Significance

Homolog detection, finding similar proteins to an unknown protein, is usually the first step in understanding the role and function of that protein. However, if the identity of protein sequences between query and target proteins is low (< 30%), traditional tools struggle to distinguish a correct match from a random one, failing to identify important similarities. We have used protein representations from deep learning language models to solve this problem. Reducing the size of these representations significantly improved homolog detection capabilities. Our tool can find putative homologs for more than 93% of human proteins that were not able to assign a function as of March 2022.

The revolution in genome sequencing has rapidly increased the volume of gene/protein sequences. However, the relationships among sequences are incomplete but critical to understanding function, evolution, and synthetic biology. Extracting meaningful relationships from the vast amount of sequence data is an important challenge. Here, significant progress is made with a method that relates protein sequences to one another for large numbers of cases previously unknown. Finding similar sequences is usually needed to understand any new sequence, and such homolog detection is one of the most important and most frequently used kinds of computations throughout biology, as evidenced by the heavy use of BLAST and its large number of citations.

## Sequence Matching Is the Most Common Way Homologs Are Identified.

Sequence similarity searches usually begin with scoring pairs of sequences. These algorithms utilize substitution scoring matrices to score all of the amino acid substitutions, usually based on the similarity of each pair of matched amino acids in the alignment. There are two types of sequence alignments: global alignments typified by the Needleman–Wunsch (1) and local alignment (2). However, exact searches can be computationally expensive to perform on a large scale because of their *O*(*n*^2^) time complexity. To speed up the search times, successful heuristic methods were developed such as BLAST (3) and FASTA (4). When sequence similarities between query and homolog proteins are high (> 30%), these methods usually perform in a completely satisfactory way. However, for so-called twilight zone proteins having lower sequence identities in the range of 25 to 30%, these methods can often fail to identify homologs (5–7). To remedy this, several variants of BLAST have been proposed. Profile alignment methods use multiple sequence alignments to generate a probabilistic model for the query protein. These profiles can then be used to perform a search on the target database. These methods also can iteratively increase the quality of the search by including into the query profile the hits that were newly found and by repeating the search. Some examples of such methods are PSI-BLAST (8) and CS-BLAST (9) tools. Another popular direction of research uses hidden Markov models (HMM). These methods can also yield a probabilistic representation of protein families and perform alignments based on hidden Markov model (HMM) representations. Tools such as PHMMER (10) or HHSEARCH (11) are based on these HMM representations. Our recent progress in sequence matching has explicitly incorporated generic structural information to improve the sequence matching for improved agreement between the sequence and structure matches (12). But, none of these methods are able to consistently identify remote homologs in the 20 to 25% sequence identity range.

## Structures Are More Conserved than Sequences.

Despite the heavy use of sequence matching by biologists, protein homolog detection is far from being solved. The fact that protein structures are more conserved than sequences makes this a difficult problem. An ancestral reconstruction showed that structures have been preserved for over 4 billion years (13), but those sequences over such a long time are highly variable. Accordingly, sequence matching alone may not be the best approach for homolog detection. Moreover, heuristics utilized in BLAST are known to yield inconsistencies in homolog detection when different e-values are used (14–16). Importantly, in a recent paper, Weisman et al. (17) showed that most genes previously believed to be lineage specific are instead cases of homolog detection failures. In their framework, it was shown that sequence similarity tools cannot distinguish between rapidly evolving genes and fully random matches, resulting in frequent homolog detection failures. This shows the limit for pairwise sequence similarity searches and how these failures can drastically affect evolutionary connections.

## Numerical Representations of Sequences.

In the meantime, machine learning methods have developed efficient ways to represent large-scale data; unsupervised learning has allowed researchers to utilize the vast volume of protein sequence data to train neural networks to generate context-aware numerical representations (embeddings) of the input amino acid sequences. These representations retain biochemical, biophysical, and evolutionary information about the input sequences. Embeddings encode remote homology, protein family information, secondary structures, tertiary contacts, and mutational effects. When compared with current predictive bioinformatics tools, neural network-generated embeddings usually can either outperform state-of-the-art methods or reach a similar level of success (18).

## Linguistic Embedding and Data Compression.

The present work is based on the powerful neural network embeddings of protein sequences from Rives et al. (18) and Raimondi et al. (19) that utilize inverse direct cosine transform (iDCT) quantizations and dynamic time wrappings (DTW) to find protein similarities. Quantization is a term for the method of reduction from high-dimensional data to a low dimension representation. Here, we develop a method named PROST that applies iDCT to the embeddings from the ESM (Evolutionary Scale Modeling) protein language model (18). Quantization has some important advantages over a larger dense layer. First, quantization does not need further training. Accordingly, we can use the whole benchmark for testing instead of splitting it into subsets for testing, training, and validation. Second, classification after quantization is significantly faster than even with the simplest neural layer. Finally, the results with our quantization already produce important large gains in performance. Quantization with iDCT results in a significantly more concise representation of sequences. Having smaller representations allows us to perform significantly more efficient searches without any heuristics. With a single-core AMD EPYC 7543 CPU, our PROST can perform a search against the SwissProt database within a second. PROST finds putative remote homologs for proteins that previously had no known related sequences. These same proteins had no remote homologs identified by traditional sequence matching tools because of their low sequence identities. Our method is not only faster but also more accurate in application to the benchmarks (20) from Pfam (21), SUPERFAMILY (SCOPe 2.08) (22, 23), and CATH Gene3D (24, 25) datasets. PROST is an alignment-free method that simply compares the embedding vectors for a high level of efficiency.

## Method

The first step is obtaining the protein representation with the ESM1b protein language model, which is a transformer-based neural network model inspired by the recent successes of transformers in the natural language processing field. The ESM1b model is trained by masking some of the residues from the input sequence and training the model to predict the masked residues. Hence, the model must learn the relationships among the residues. It is trained on the UniParc database from UniProt (26) with 250 million sequences. The model can distinguish amino acid types by their biochemical properties. Although no evolutionary information is input, the model learns the family information from the sequences. The model can predict biophysical properties such as secondary structures and contacts between residues. Moreover, a variant of it can predict mutational effects (27). These results provide strong evidence that embeddings with the ESM1b protein language model can also be applied for homology detection. However, the embeddings contain extraneous information for homolog detection since these same embeddings can also be used to predict multiple properties. Results are improved by quantizing the embeddings to compress the data and retain the most essential information for homolog detection. When no quantization is applied and raw embeddings from ESM1b are used in homolog detection with the help of the dynamic time-wrapping technique (28), we find that the accuracy by the AUC metric is 7.4% worse compared to the compressed version, supporting evidence that the uncompressed embeddings have more information than is required.

### Highly Efficient Global Representations of Proteins.

**Fig. 1.**
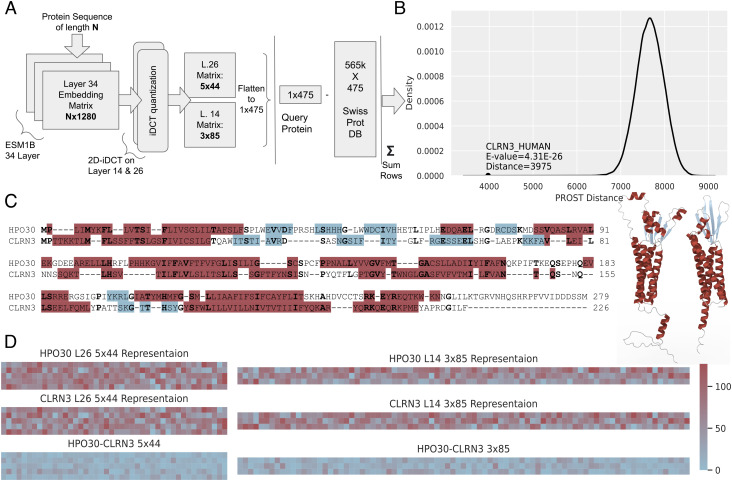
The PROST architecture and parameter optimization. (*A*) The PROST architecture. A protein sequence is fed into the ESM1b language model to obtain embeddings that are reduced to maximize accuracy for remote homolog detection. Accordingly, two representations are carried through, with every protein being represented by two different matrices for two different compression levels, chosen during optimization (*SI Appendix*). (*B)* An example—a PROST search for HPO30 protein (having no previously known human homolog) against the SwissProt database. The PROST distance distribution is similar to a normal distribution for nonhomologs. Putative homologous proteins are outliers in this distribution. Robust z-scores with Bonferroni corrections are used to calculate the expectation values of randomly finding such a homolog. The CLRN3 protein was found as a putative homolog for HPO30. (*C*) Sequence alignment and structures of HPO30 and CLRN3, showing validation by similarity in structures. Global alignment with the ProtSub matrix (12) yields a 21.5% sequence identity. Helixes are colored red; beta sheets are colored blue. Structures are from AlphaFold2 predictions (29), with HPO30 on the right and CLRN3 on the left. (*D*) Visualizations of PROST representations for HPO30, CLRN3, and the differences between them. The sum of all elements in the difference matrices gives the PROST distance, in this case, 3975 as shown in part b.

Direct cosine transform (DCT) is widely used for lossy compression resembling those used in the JPEG format. It has been used in protein–protein interaction prediction (30), secondary structure prediction (31), and protein folding (32). Similarly, the Fourier transform was used in the representation of the torsional energies of the amino acid bonds (33). The iDCT quantization method was introduced recently (19), and it has properties that are particularly useful for sequence representation. First, it preserves the sequential nature of the embeddings. Second, after quantization, it retains most of the input information. 2D-iDCT is similar to image compression in reducing both input dimensions of a given matrix. For a protein with N residues, the ESM1b embedding will be in a 3D matrix having dimensions 34 × N × 1,280, where 1,280 is the embedding length of a residue and 34 is the number of output layers of the language model. Each layer of ESM1b consists of 20 attention heads. Every attention head learns a different relevance of the input sequence. It was shown that natural language models based on transformers learn to attend to direct linguistic features such as objects of verbs, qualifiers of nouns, and more. Moreover, they showed that attention heads in the same layer perform similar jobs (34). For example, an attention head in a protein language model may look for disulfide bonds between cysteine residues. However, what every attention head learns is unknown beforehand and a special dataset, training, and testing are needed to figure this out. So, we perform an optimization to find out which layer, along with the compression ratio, result in the best homolog detection performance. We select an individual layer and apply 2D-iDCT to reduce it. These dimensionality reduction methods are analogous to principal component analysis and act to remove noise. The optimization study indicates that layer 26 with 5 × 44 and layer 14 with 3 × 85 have the best accuracies with the smallest memory footprint. The intuition behind having two matrix representations for every protein is to include different levels of quantization with the different layers to increase the representation capability. Representations for every query protein and every protein in the database are all precomputed and generated in advance. To compare two protein sequences, it is necessary to take only the sum of the absolute differences between every element in the two sets of representation matrices. If the two proteins are homologs, the sum of these will be is small. This reduction significantly speeds up the search operation. PROST searches have linear time complexity (*SI Appendix*, Fig. S2). The algorithm is highly parallelizable and uses vector operations. A single protein is represented by just 475 bytes. The detailed architecture of PROST is shown in Fig. 1*A*. (*SI Appendix* for some further details, particularly for the optimization of the compression parameters). We note that the compression parameters of PROST are chosen to minimize the memory requirements and to perform better than state-of-the-art methods. However, with a larger more complete representation, it is possible to achieve even better results. (*SI Appendix* shows those results as PROST-L). PROST uses a fixed-length (variable-ratio quantization) representation of proteins. Combined with simple distance calculations, this allows the fastest possible searches. However, this feature undermines the ability to detect local homology. Local fragments are not usually detected well by PROST.

### Statistical Evaluation of Protein Homolog Distances.

In a traditional bioinformatics workflow, a query protein is searched against a large protein database. For this query protein, PROST will calculate a distance for each target protein in the database, resulting in a distribution of distances. This distribution is usually similar to a normal distribution. Putative homologs to the target protein will have better scores and be outliers in such a distribution. An example distribution is shown in Fig. 1*B* for the distances between the query protein, HPO30, and its scores against all SwissProt proteins. From the distribution of these scores, we can then calculate the robust z-scores and the probability compared to the cumulative distribution function to learn whether a protein has a statistically significant score. The calculated value will be the probability of having a nonhomologous pair with a given distance score. These are similar to the expectation values provided by BLAST. Robust z-scores normalize the distribution by using medians instead of means and median absolute deviations (MAD) instead of standard deviations (35) to be more effective for outliers.

In the case of a search with a member of a big protein family, putative homologs may not be outliers. They might be the majority of proteins in the database. In such a case, probable homologs may be first filtered out before e-value calculation. This can be done using the PROST distance threshold that is found by maximizing the F1 score at the max50 dataset. Distance values smaller than this threshold will not be used in the calculation of mean and SD. This way, the normality of distance distribution of nonhomologs will be preserved even with the high number of putative homologs. Lastly, e-values can be calculated for the whole database using the mean and SD found for nonhomologs.

### An Example of a Homolog.

HPO30 is used as an example to demonstrate the PROST pipeline. It is an uncharacterized protein of *C. elegans*. NCBI-BLAST and PHMMER do not find any human homologous sequence for HPO30 in the SwissProt database. However, it is known that this protein belongs to the Pfam claudin-like domain (PF07062). When searched against SwissProt, PROST quickly identifies hundreds of putative homologs to HPO30 that have around 20% sequence identity. The PROST distance distribution of SwissProt for this protein is shown in Fig. 1*B*. Nonhomologs follow a normal distribution while putative homologs will be outliers to this distribution. Outliers can be found using z-scores with Bonferroni multiple test correction. One example of alignment with the putative human homolog CLRN3 (Q8NCR9) is shown in Fig. 1*C*. HPO30 and CLRN3 have 21.5% sequence identity; however, they have the same structure fold. Structures of the two AlphaFold2 (29) predictions for these proteins are shown in Fig. 1*C*. Overall, the core structure alignment has an RSMD of 3.4 Å. Fig. 1*D* shows the PROST representation for HPO30, CLRN3, and their differences.

## Dataset and Validation

In the evaluation of our method, we use a benchmark set (20) which contains three different datasets from Pfam (21), Gene3d (24), and SUPERFAMILY (22). These datasets contain proteins with multiple domains. If two proteins have the same sequential domain order, they are indicated as homologs. If they do not share any domain in common, then they are annotated as nonhomologous. The benchmark is divided into two sets. The first set, which limits the lengths of regions between domains to 50 residues, we named “max50,” can be seen as a global homology benchmark. The second set includes the others without this limitation, which we named “nomax50.” In the “max50” benchmark, the Pfam dataset contains 5,245 homolog pairs. The Gene3d dataset has 5,047 such pairs. The SUPERFAMILY contains 5,656 pairs. Each dataset also contains the same number of nonhomolog pairs as homolog pairs. The total number of protein pairs that are included in the max50 benchmark is 31,896, while the nomax50 benchmark has 180,566 pairs. The benchmark dataset was curated from 16 species using domain databases instead of structural databases to make sure that pairs of proteins of unknown structures have also been included. Even though the curation method is different for each database, they have only a 0.1% disagreement regarding whether a protein pair is a homolog or not. This demonstrates the unequivocal agreement within the parts of the benchmark dataset (20). We compare the PROST accuracy with the same metrics used in the benchmarking paper (20). Specifically, the area under the ROC curve calculated on the first 1,000 false positives (AUC1000) is used here as the metric for performance.

## Results

The evaluation of the PROST tool has been carried out against the most commonly used alignment-based homolog detection tools. The Pfam, Gene3D, and SUPERFAMILY datasets are used for testing. The AUC1000 score indicating the quality of the ranking is used to judge performance. We have compared against CSBLAST (9), PHMMER (10), NCBI-BLAST (3), and FASTA (4) scores reported in the previous benchmarking paper (20). Moreover, we also compared PROST with the homology detection method used in the ESM1b paper (18). In that method, the mean of the last (34th) layer of ESM1b embedding columns is used as a fixed-size representation of a protein. Then, the simple distance between these representations is used as a homology indicator. This result is shown as ESM1bL34M (*SI Appendix*). PROST performs significantly better than other tools. The largest extent of improvement is a significant 9.8% gain, e.g., NCBI-BLAST compared with PROST for the SUPERFAMILY dataset.

**Fig. 2.**
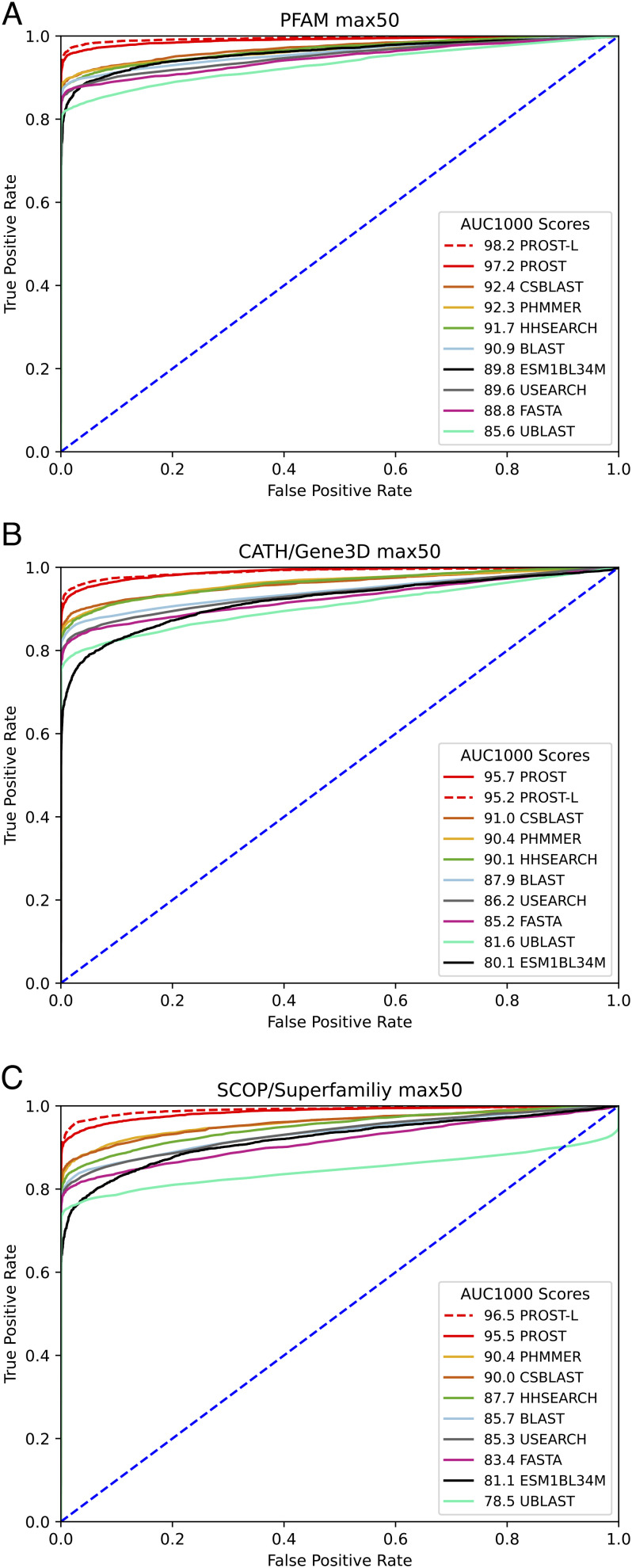
ROC plots for max50 benchmarking dataset. This dataset contains proteins with a limitation of 50 undefined amino acids in between the defined regions, constraining the homology test to global homology detection. The plots show the overall performance of tested methods as true-positive and false-positive rates. We ranked each curve based on their performance on the first 1,000 false positives measured by the AUC1000 metric. ROC plots for each database, Pfam (*A*), Gene3d (*B*), and Superfamily (*C*) are shown separately. Overall, PROST is the best tool in this dataset producing the highest AUC1000 scores. This clearly demonstrates the importance of the optimization that has been developed here by comparing the ESM1b results against the optimized PROST results.

Table 1 shows the performance of the most commonly used tools with the benchmarking dataset. It shows that PROST is the best tool with the AUC1000 (area under the ROC curve for the first 1,000 false positives) metric. PROST has a 2% higher AUC score and a 4.8% higher AUC1000 score, in comparison with the closest tool, CS-BLAST. Compared to the commonly used NCBI-BLAST tool, PROST has 3.1% higher AUC and 6.3% higher AUC1000 scores. When benchmarking is performed with the Gene3D database, PROST outperforms CS-BLAST with a 2.6% higher AUC score and 4.7% higher AUC1000 score. Lastly, in the SUPERFAMILY dataset, PROST is the best tool with a 2% higher AUC score than CS-BLAST, and in terms of AUC1000, a 5.2% higher score compared with PHMMER (PHMMER has a higher AUC1000 score than CS-BLAST). *SI Appendix*, Table S2 shows the AUC and AUC1000 metrics for all selected commonly used tools. Fig. 2 shows the ROC curves for the methods we benchmarked in the max50 dataset. These results strongly suggest that PROST is an accurate homology detection tool collectively outperforming these commonly used alignment-based homolog detection tools for the Pfam, Gene3D, and SUPERFAMILY datasets with a compressed minimal optimized data representation. These consistently permit identifying important putative homologs at lower levels of sequence identity.

**Table 1.** Comparison of homolog detection methods in the max50 dataset

	Pfam		Gene3D		SUPERFAMILY	
Method	AUC	AUC1000	AUC	AUC1000	AUC	AUC1000
**PROST**	**99.0**	**97.2**	**98.9**	**95.7**	**98.5**	**95.5**
CSBLAST	97.0	92.4	96.3	91.0	96.1	90.0
PHMMER	96.4	92.3	96.2	90.4	95.9	90.4
NCBI-BLAST	95.9	90.9	94.4	87.9	93.7	85.7
ESM1bL34M	96.0	89.8	91.9	80.1	92.0	81.1
FASTA	94.6	88.8	93.2	85.2	91.9	83.4

Table 2 shows the confusion matrix for PROST in the max50 benchmarking dataset. Moreover, *SI Appendix*, Fig. S3 shows the distribution of the homology predictions based on protein pair size differences. Overall, PROST has 1,239 incorrect predictions out of 31,896 pairs, which is a remarkably high level for correct predictions. The number of false negatives is 207, while the number of false positives is 1,032. Having lower false negatives and higher false positives may be desirable to a certain extent since a second step could reevaluate the false positives as follows: For example, a search with the SwissProt database requires over half a million comparisons. PROST can be used first to reduce the search space from millions to hundreds, and then, computationally intensive structure predictions and structural comparisons could be used to reduce some of the false positives if their structures do not match well.

**Table 2.** Confusion matrix for PROST in the max50 benchmarking dataset

	Prediction	
Total: 31896	Positive	Negative
**Actual**		
Positive	14916	207
Negative	1032	15741

*SI Appendix*, Table S3 shows the results for the nomax50 benchmarking dataset. *SI Appendix*, Fig. S4 shows the ROC curves for the methods we tested in the nomax50 dataset. We found that PROST performs mediocre for this benchmark, which signifies the global alignment nature of PROST. However, PROST-L, the version with less reduction in embedding information, has a good performance comparable to state of the art methods. PHHMER performs best on the nomax50 benchmark. It is followed by CSBLAST and PROST-L. PROST is similar to BLAST in performance, and the method that uses the mean of only the last layer (ESM1bL34M) performs the worst. To exemplify the global alignment nature of PROST, we have searched human zinc finger protein 268 (Q14587) with PROST and BLAST on the SwissProt database. BLAST found 1605 putative homologs, while PROST found only 560 putative homologs. This signifies the global alignment nature of PROST. The results are presented in *SI Appendix*, Dataset 1.

## Runtime Requirements

Every PROST search begins with the embedding process and quantizing the input sequences, i.e., the preprocessing. PROST can create a database of 100 sequences randomly sampled from SwissProt within 13.15 s ± 60 ms and 1,000 sequences within 71.89 s ± 347.8 ms using one GPU. PROST needs to create a database only once, and then, the database can be used over and over. Searching a database of 100 sequences against itself takes 519 ms ± 6.3 ms, and 1,000 sequences against itself takes 976 ms ± 10.6 ms. The nonlinear time difference is caused by differing startup overheads and disk accession delays. Embedding and searching a sequence in the preprocessed SwissProt PROST database takes only 1.02 s ± 7.3 ms. These tests have been carried out ten times to obtain the error estimates. These were a Nvidia A100 40GB GPU and a single-core AMD EPYC 7543 processor. Database creation runs faster on GPUs. However, it can also run on a CPUS. Search operations use only one core CPU.

## Discussion

The method of homolog detection in PROST is extremely different from traditional sequence or PSSM-based profile alignment tools. We have investigated the effects of this difference for its ability to identify homologs as well as on the performance by using different metrics. In a recently published paper (17), it was shown that as the rate of protein evolution increases, the probability of remote homolog detection decreases for the usual sequence similarity search tools. This happens because the sequences ultimately are so diverged that existing tools cannot distinguish them from random matches. Importantly, the framework from Weisman et al. (17) can predict for a gene whether the rate of evolution is too high for remote homolog detection with traditional sequence alignment-based tools. In their study, they found that most genes with no detected homologs outside of the specific lineage are wrongly attributed to biological novelty. They concluded that the majority of genes identified as being lineage specific are instead a result of homolog detection failures.

Intrigued by this, we performed PROST searches on lineage-specific *S. cerevisiae* genes, where there are a total of 325 lineage-specific genes, 155 of them with at least three homologs in the lineage so that the evolutionary rate can be calculated (17). Of these, 126 are predicted to evolve rapidly and, consequently, might not be detectable in out-of-phylum species. We performed PROST search on 7 out-of-phylum species, *E. gossypii, K. lactis, N. castelli, L. waltii, Y. lipolytica, and A. nidulans, S. pombe*, 4 of which, *E. gossypii, K. lactis, N. castelli, and L. waltii*, had synteny information on the Yeast Gene Order Browser (36) database. PROST found significant hits for 73% or 92 out of the 126 genes, while a synteny-based BLAST search found homologs for only 8.7%, or 11 genes, with agreement between the PROST and BLAST synteny results for 72.7% or 8 genes. Synteny-based searches are more sensitive because only the gene in the ortholog’s chromosomal locus is tested for homology instead of the whole proteome, removing the burden of multiple testing corrections, and this provides increased sensitivity. Even though the PROST search was done for the 7 genomes with Bonferroni correction, it found significant hits for three times more genes with the same e-value 0.001. For the remaining 29 genes in yeast, the lineage specificity cannot be explained by the rate of evolution, PROST does find putative homologs for 72.4% or 21 genes, whereas the synteny-based BLAST search found homologs only for 41.4% or 12 genes, and agreement between the two was seen for 66.6% or 8 genes. These results indicate that PROST can find putative homologs for lineage-specific genes that are evolving too fast to be detectable with usual search methods; it is significantly more sensitive than synteny-based searches with traditional tools. The larger numbers of hits found suggest that this method may have some potential for overcoming well-known errors in the annotations, since larger numbers of putative homologs that are found to have the same function should increase the reliability of these functions identified from sequence matches. Results are presented in an interactive website https://mesihk.github.io/prostyeast.

**Fig. 3.**
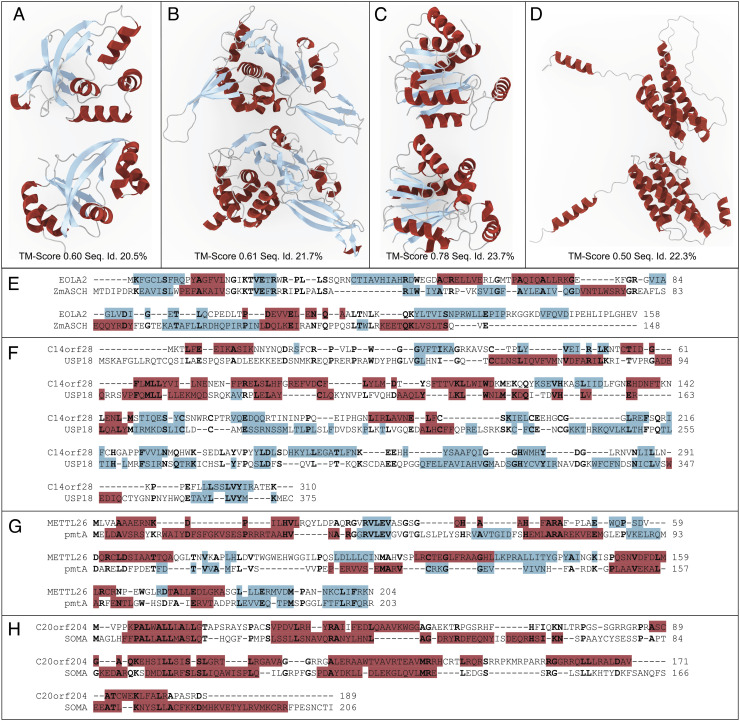
Examples of putative homolog prediction by PROST, validated by structural similarity. Several cases of human proteins that presently have no current GO annotations (*Top* structure in each box) together with hits found by PROST (*Bottom*). Structures are AlphaFold2 (29) predictions unless otherwise noted. Structural alignments were done with the TM-Align tool. A TM-Score ≥ 0.5 indicates the same structural fold (38). Sequence alignments are done using the ProtSub substitution matrix (12) with a gap opening penalty of 5 and extension penalty of 1. Identical residues are shown in bold. Helixes are colored red; beta sheets are colored blue. (*A*) Human EOLA2 and *Zymomonasmobilis subsp.* ASCH domain-containing ribonuclease (PDB ID: 5GUQ). (*B*) Human C14orf28 and *Pongo abelii* Ubl carboxyl-terminal hydrolase 18. (*C*) Human methyltransferase-like 26 and *Cereibacter sphaeroides* phosphatidyl ethanolamine N-methyltransferase. (*D*) Human chromosome 20 open reading frame 204 (C20orf204) and *Protopterus annectens* Somatotropin. (*E*) Alignment of Human EOLA2 and *Zymomonasmobilis subsp.* ASCH domain-containing ribonuclease proteins. Sequence identity is 20.5%. (*F*) Alignment of Human C14orf28 and *Pongo abelii* Ubl carboxyl-terminal hydrolase 18 proteins. Sequence identity is 21.7%. (*G*) Alignment of human methyltransferase-like 26 *Cereibacter sphaeroides* phosphatidylethanolamine N-methyltransferase proteins. Sequence identity is 23.7%. (*H*) Alignment of human C20orf204 and somatotropin. Sequence identity is 22.3%.

Motivated by this, we investigated all human proteins that are in SwissProt and as of March 2022 had no GO functional annotations assigned. We found 864 such proteins. BLAST and PROST are used with an e-value of 0.05 to find homologous proteins to these sequences. FATCAT, a tool that aligns protein structures with twists and rotations, is used to obtain the structural similarity significance (37). We find PROST results to be more informative. PROST had statistically significant structural hits for 73.8%, 628 human proteins, but BLAST found only 58%, or 494 human proteins. We investigated some of the cases where PROST had significantly more hits than BLAST. Fig. 3*A*–*D* shows four human proteins that presently have no assigned GO annotations. The hits found by PROST for these proteins are all structurally similar. All of these sequence and structural alignments are given at https://mesihk.github.io/prosthuman.

In the breakdown of the most significant hits for unknown human proteins, PROST and BLAST together found the best putative homologs for 40.2% of human proteins, while 26.3% were found only by PROST and 9.9% found only by BLAST. The remaining 23.6% had not identified structurally verified homologs. All in all, 76.4% of human proteins had putative homologs with significant structural alignments, of which 9% had uncharacterized functions.

**Fig. 4.**
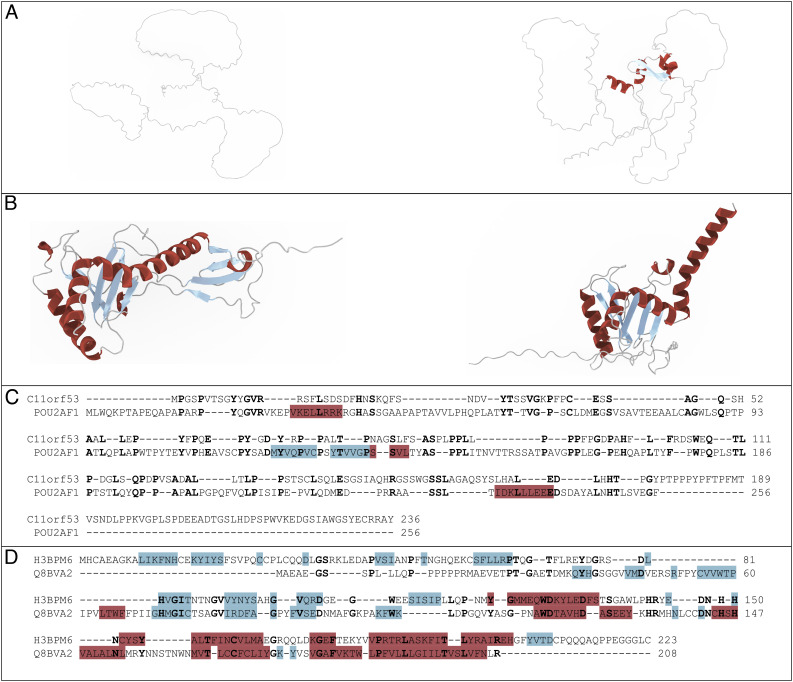
Two example case with no clear structural similarity (*A*) and a case with low sequence identity (*B*). Human chromosome 11 open reading frame 53 (C11orf53) was an uncharacterized protein in March 2022. A recent paper (39) experimentally showed that it is a transcriptional coactivator of POU2F3 and plays a role in the generation of Tuft cell lineage. The putative homolog found by PROST is POU2AF1, which is a transcriptional coactivator that associates POU2F1 and POU2F2. C11orf53 has a similar function with the POU2AF1 found by PROST, validating the homology relationship. PROST identifies a putative homolog for human MKRN2 opposite strand protein (H3BPM6) with a 16.4% sequence identity. (*A*) Structures predicted by AlphaFold2 (29) for C11orf53 (*Left*) and POU2AF1 (*Right*) show no clear similarity. (*B*) Structures predicted by AlphaFold2 (29) for H3BPM6 (*Left*) and Q8BVA2 (*Right*). The alignment of these structures has a FATCAT p-value of 2.91e-03 and a 0.47 TM-Score. (*C*) Sequence alignments of C11orf53 and its putative homolog POU2AF1 have 22.2% sequence identity. (*D*) Sequence alignments of human MKRN2 opposite strand protein (H3BPM6) and Q8BVA2 have 16.4% sequence identity.

This large number of predictions is also useful for learning the false-positive rate of PROST. Of 864 human proteins, 14 did not have any AlphaFold predictions. The remaining 850 proteins had a total of 44,444 hits found by PROST. However, 6,645 hits did not have AlphaFold predictions. The rest of the 37,799 hits were structurally aligned using the FATCAT tool (37). This tool applies twists and rotations to match given structures and reports a statistical significance as a p-score. Out of 37,799 PROST hits that are found for a 0.05 PROST e-value cutoff, only 31.5% did not have a significant structural alignment. When the PROST e-value cutoff is more stringent, at 0.001, among 20,672 hits, the ones having no significant structural alignment are reduced to 22.2%. These are rough false-positive estimates for PROST. But as showcased in Fig. 4*A*, a PROST hit may have a similar function even though they do not have any clear structural similarity. One can expect 1/3 of hits to have no structure similarities when using an e-value threshold of 0.05; similarly, nonstructural hits are 1/5 for an e-value cutoff of 0.001. The same analysis has been applied to BLAST. When the BLAST e-value cutoff is 0.05, the hits producing no significant structural alignments were 15.6%, and for a BLAST e-value cutoff of 0.001, this ratio falls to 13.4%

A protein with PROST hits was the human chromosome 20 open reading frame 204 (C20orf204). For this protein, BLAST and PHMMER fail to identify homologous sequences other than the mouse C20orf204 variant, while PROST identifies 731 such sequences. It was recently shown that C20orf204 is associated with hepatocellular carcinoma (HCC) (40). This protein interacts with nucleolin and ribosomal RNA and results in increased protein synthesis in the cell. C20orf204 is specifically expressed in HCC and associated with tumor development. The protein is mainly detected in the nucleus. HeLa cells overexpressing this protein grow twofold faster. C20orf204 is thought to play a role in nucleolin stabilization, resulting in increased rRNA transcription (40). Putative homologs found by PROST are mostly interleukin variants and growth hormones. Fig. 3*A* shows the structural alignment of C20orf204 and somatotropin (growth hormone) of *Protopterus annectens*, and Fig. 3*E* shows the sequence alignment. It is known that somatotropin increases the number of ribosomes (41). It also increases the protein content of muscle which makes it widely used in animal farming. C20orf204 has a similar structure to somatotropin but a vastly different sequence. However, these two proteins likely function similarly. This similarity poses an important experimental challenge of whether overexpressed somatotropin has the same effect on HeLa cells as C20orf204. There will certainly be many similar such discoveries in applications of this method.

Of note, 51% of the whole human proteome is considered to be intrinsically disordered proteins or proteins with disordered regions (42). Accordingly, most of the unannotated human proteins we studied with PROST are in this category. Disordered regions do not have clear structures. PROST hits found for this type of protein often do not have clear structural agreements. One human IDP we studied with PROST, human chromosome 11 open reading frame 53 (C11orf53), did not have any annotation as of March 2022, but later, it was studied experimentally and assigned a function (39) creating a case for PROST homolog verification. C11orf53 was found to be a transcriptional coactivator of POU2F3 and critical for Tuft cell lineage (39). Remarkably, the PROST hit for this protein was POU2AF1. There is no clear structural agreement either with tools TM-Align (38) or FATCAT (37) since both proteins lack structured regions. Structural comparison is shown in Fig. 4*A*. Moreover, the sequence identity is just 22.2% as shown in Fig. 4*B*. However, POU2AF1 is also a transcriptional coactivator that associates with POU2F1 and POU2F2 verifying homology relationship. This shows that PROST has the potential to find putative homologs for intrinsically disordered proteins that share similar functions even without significant structural similarity.

PROST can identify hits with very low sequence identity, and this represents major progress in putative homolog detection. An example case with only 16.4% sequence identity is shown in Fig. 4*D*. Similarly, structures are shown in Fig. 4*B*. These structures are significantly similar with a FATCAT *P*-value of 2.91e-03 but have a TM-Scoreof 0.47.

## Conclusions

Here, we have presented PROST, an exact search-based, extremely fast and accurate homolog detection tool that utilizes a protein language model. It is a significantly different method from other search methods in its alignment-free and sequence similarity-free approach that relies upon reduced protein representations, enabling it to perform huge numbers of comparisons within a short time. PROST outperforms all commonly used traditional alignment-based tools on benchmarks taken from three databases—Pfam, Gene3d, and SUPERFAMILY. PROST efficiently uses memory and can keep hundreds of millions of protein representations in the memory of a server-grade computer. PROST is not limited to the usual limits of sequence similarity-based tools. It can find putative remote homologs for proteins that have a high evolution rate that makes them completely indistinguishable from noise with traditional alignment-based tools, and these results push sequence alignment well below the usual twilight zone. This is exemplified by human proteins that presently do not have any annotations. PROST finds hits with highly similar structures at lower levels of sequence identity, which act to validate the sequence homologies. The recent advances in structure predictions (29, 43, 44) allow us to find support for the predicted homologs for cases where there are no experimental structures. The remarkable speed gains by PROST will facilitate rapid whole-proteome or whole-genome-scale analyses which will aid in generating more accurate phylogenies and can be important in improving our understanding of evolution and disease mutations. Also, the greater sensitivity of PROST suggests that it might be better at distinguishing the effects of mutations.

## Supplementary Material

Appendix 01 (PDF)

Dataset 01 (XLSX)

## Data Availability

The software and data are available in GitHub repository: https://github.com/MesihK/prost.
