# Supplementary material for: Improved global protein homolog detection with major gains in function identification
Source: Proc Natl Acad Sci U S A. 2023 Feb 24;120(9):e2211823120. doi: 10.1073/pnas.2211823120 (PMC9992864; doi:10.1073/pnas.2211823120)
Supplement: Supplementary file 1 — Appendix 01 (PDF) [file pnas.2211823120.sapp.pdf]

Supplementary Information for

Improved Global Protein Homolog Detection with Major Gains in Function Identification

Mesih Kilinc, Kejue Jia, and Robert Jernigan

Corresponding author Robert Jernigan.
E-mail: jernigan@iastate.edu

This PDF file includes:

Supplementary text
Figs. S1 to S4
Tables S1 to S3
Legend for Dataset S1
SI References

Other supplementary materials for this manuscript include the following:

Dataset S1

Supporting Information Text

Distance Metric Selection. PROST represents proteins in a small, compressed embedding matrix. To find out the homology of two given proteins, we calculate the distance between the compressed embedding matrices. We evaluated different distance metrics. Table S1 shows the area under the curve scores for the prediction of PFAM (1) protein pairs that are taken from the max50 benchmarking dataset (2). In this test, 5x44 quantization of the 34th layer is used as a compressed representation of given proteins. We selected the L1 distance metric because it is cheap to calculate and gives the best result after the dynamic time wrapping (DTW) method (3).

Compressed Protein Representation Optimization. There are several objectives in the choice for the protein representation:

- Capturing the most essential information from the embeddings. Surprisingly, when no compression is applied to either dimension (Layer 34, N× 1280), the accuracy for the Pfam dataset given by the AUC metric is already 91.6%, only 7.4% lower than the selected compression scheme (Layer 26, 5 × 44 and layer 14, 3 × 85).

- Reducing the size of the database, so it will fit into RAM memory, for faster search times. Our goal is to fit the NCBI non-redundant database into commodity server-grade memory.

- A small number of columns in the protein representation matrix won't need alignment so that the distance will be sufficient for searching and scoring.

ESM1b language model has 34 output layers. Fig. S1-a shows the performance for different layers of the ESM1b. The first layers perform poorly. However, the best performing layer is not the last layer (34), but instead, it is layer 26. We found that using two different layers with two different compression levels increases the performance considerably. Next, we tried to find best compression ratio. Fig. S1-b shows the heatmap of AUC scores with different parameter selections with the distance metric. We used AUC for the PFAM dataset when the 34th layer of ESM1b is used with different compression ratios. The X-axis shows the number of columns we preserve in the embedding, and vice versa Y-axis shows the number of rows compressed embedding has. There it can be seen that as the number of columns increases, the distance metric performs poorly since we are nearer to a full residue representation. However, a higher row count increases the AUC score as more information is available for the comparisons. Nevertheless, high dimensionality increases the demand for memory, resulting in database fragmentation leading to substantially longer search times. DTW method is better than other distance metrics because it is similar to global alignment. We wanted to investigate the effects of alignment (DTW) on the compressed representations. Fig. S1-c shows the difference between the L1 and DTW metrics in predicting homology. We saw that when the number of columns is low (<10), then the difference between the two methods is negligible. However, the DTW method has $O(n^2)$ time complexity, while the L1 method can be computed in $O(n)$. Due to this, we used L1 distance in the PROST pipeline. Finally, a compression scheme that uses layer 26 embeddings with 5 columns and 44 rows and layer 14 embeddings with 3 columns and 85 rows is the best option to have state-of-the-art accuracy without needing actual alignments. The memory footprint for each protein is a humble 475 bytes resulting in easily manageable database sizes.

While having a low column count in protein representation allows us to use the L1 metric instead of alignment, having a high number of rows allows us to represent proteins more completely. We tried different compression ratios and found if we use a 3x1280 compression ratio with layers 14 and 26, we can maximize the prediction performance, albeit at the cost of a bigger representation. We termed this bigger representation as PROST-L. Compared to PROST, PROST-L has 16 times bigger protein representation while only minimal performance increase in the max50 benchmark. Table S2 shows that PROST-L has 99.3% AUC for PFAM pairs while PROST has 99% AUC, only a 0.3% difference. However, PROST-L performs considerably better than PROST in the nomax50 benchmark but not as good as PHMMER. Due to this, we selected a smaller representation with minimal accuracy loss to maximize the PROST pipeline's speed and focused on detecting global homologs.

Statistical Significance Test in PROST Pipeline. PROST generates distances for a query protein against every protein in a database. These distances will form a distribution. Any query protein may have hundreds of homologs (outliers with small distances in the distance distribution) within a database of more than half-million proteins. This makes using a z-score quite effective in judging similarities and differences when there are multiple protein homologs identified by PROST. Robust z-scores normalize the distribution by using medians instead of means and median absolute deviations (MAD) instead of standard deviations (4). A multiple test p-value correction should be applied because we test homologies for thousands or even millions of proteins. In the PROST pipeline, we use robust z-scores with Bonferroni multiple test corrections (5). Accordingly, a cut-off value (ex: 0.05) can be used for the expected values to find good homologs within the database.

PROST Has Linear Runtime. PROST searches have linear time complexity that depends on the size of the target database. Fig. S2 shows empirical test results with different database sizes used to query a protein. Time requirements increase linearly. The exact search time for SwissProt (6) database is just 1.02 s ± 7.3 ms which ∼ 0.65 s of it is for the startup overhead and embedding the query sequence and ∼ 352 ms is the actual search time with a single-core AMD EPYC 7543.

PROST is the Best Tool in the max50 Benchmark Dataset. Table S2 shows the AUC and AUC1000 scores of common homolog detection tools. PROST has the best results on all 3 datasets. PROST-L has slightly higher scores with a 16-fold more memory requirement.

PROST is a global homology detection tool. Table S3 shows that PROST only have mediocre performance on the nomax50 dataset which focuses on the local homology detection due to the unlimited undefined regions between defined domains. PROST-L compared to PROST has considerably high performance but not as good as current tools. PROST performs close to level of NCBI-BLAST (7) and the method that only uses the mean of the last layer (ESM1bL34M (8)) performs the worst. Moreover, Fig. S3 shows the predicted outcome of protein pairs with the differences in sizes between query and target proteins in the max50 dataset. It can be seen that as the size difference increases PROST performance decreases. PROST is clearly better at whole protein homolog identification. To exemplify PROST performance on a big and diverse protein family, we have searched Human Zinc finger protein 268 (Q14587) with PROST and BLAST on the SwissProt database. BLAST found 1605 homolog while PROST only found 560 homologs. High number of homologs may not be found by outlier detection method PROST currently uses. Accordingly, we performed statistical significance test on PROST results by first filtering probable homologs by distance threshold and then calculating the mean and median for statistical significance test. With that we were able to find 3 more sequences, totaling to 563. This test showcases the global alignment nature of PROST and presents the local alignment weakness. The SwissProt database contains more than 1/2 million sequences. Accordingly, the newly purpsed e-value calculation doesn't have a major impact. We compiled this result and presented at the SI dataset 1.

Threshold Selection Based on Benchmark Results. Benchmarking results in the max50 dataset can be used to select a threshold for the distance metric to identify homologs and non-homologs. Using the max50 benchmarking dataset, we have calculated F_1 scores for different thresholds and selected 6828.5 since it maximizes the F_1 score.

$$F_1 = 2\frac{\text{percision} \times \text{recall}}{\text{percision} + \text{recall}} = \frac{\text{tp}}{\text{tp} + \frac{1}{2}(\text{fp} + \text{fn})} \qquad [1]$$

Distance threshold is useful in several ways. First, it can be used for single comparisons of two proteins. If the PROST distance of these two proteins is lower than the threshold they might be homologs. Secondly, the PROST distance is related to the phylogenetic distance. Accordingly, PROST distance itself is not only useful for classifying homologs but also useful for measuring the closeness of protein pairs.

PROST on Unannotated Human Proteins. We used PROST on human proteins that are in the SwissProt database and have no GO annotation as of March 2022. There are 864 such proteins and 851 of them had Alphafold2 (9) structure predictions. BLAST and PROST are used with an e-value of 0.05 to find homologous proteins to these sequences. FATCAT (10), a tool that aligns protein structures with twists and rotations, is used to get structural similarity significance for all of the homologs that had Alphafold2 predictions. We find PROST results to be more informative. PROST had statistically significant structural hits for 73.8%, 628 of human proteins, but BLAST found for only 58%, 494 of human proteins. Sequences are aligned with the ProtSub (11) matrix using a gap opening 5 and a gap extension 1 penalties. We took the best structurally aligned homolog and made a sequence alignment. Most of these proteins were intrinsically disordered proteins. Accordingly, they have poor structural alignment, but the overall protein shapes are similar between query and PROST homolog. Results are given at mesihk.github.io/prosthuman.

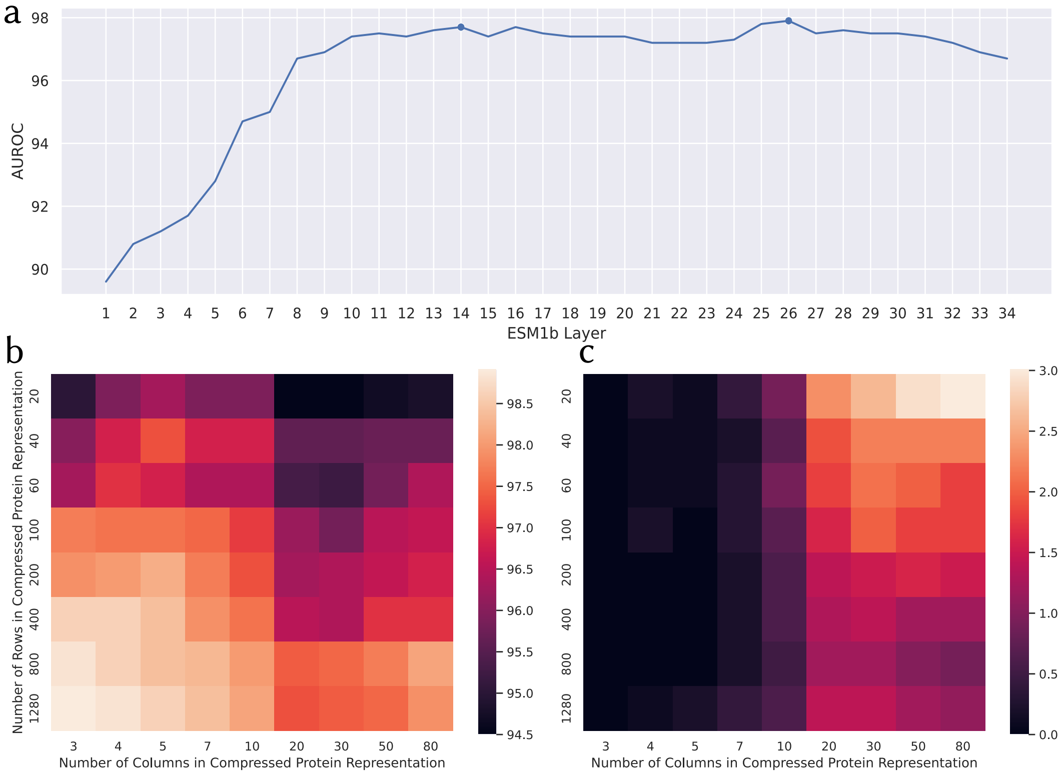

Fig. S1. PFAM AUC Score difference between DTW and L1 metrics when different compression ratios are used. a) Overall prediction performance over the whole benchmarking dataset when different ESM1b protein language model layers are used with only 5 × 44 quantization. Layer 26 has the best prediction capability. PROST uses layer 14 coupled with layer 26 to get the best accuracy. **b)** Effect of different embedding compression ratios on PFAM AUC score. The lower column count has good prediction capability due to not requiring alignment. A higher row count increases the score with the expense of memory footprint. **c)** The difference in the AUC scores between the distance and the DTW technique (global alignment) is shown. When the column dimensionality is below 10, the differences are negligible. The distance calculation has a time complexity advantage compared to the DTW method.

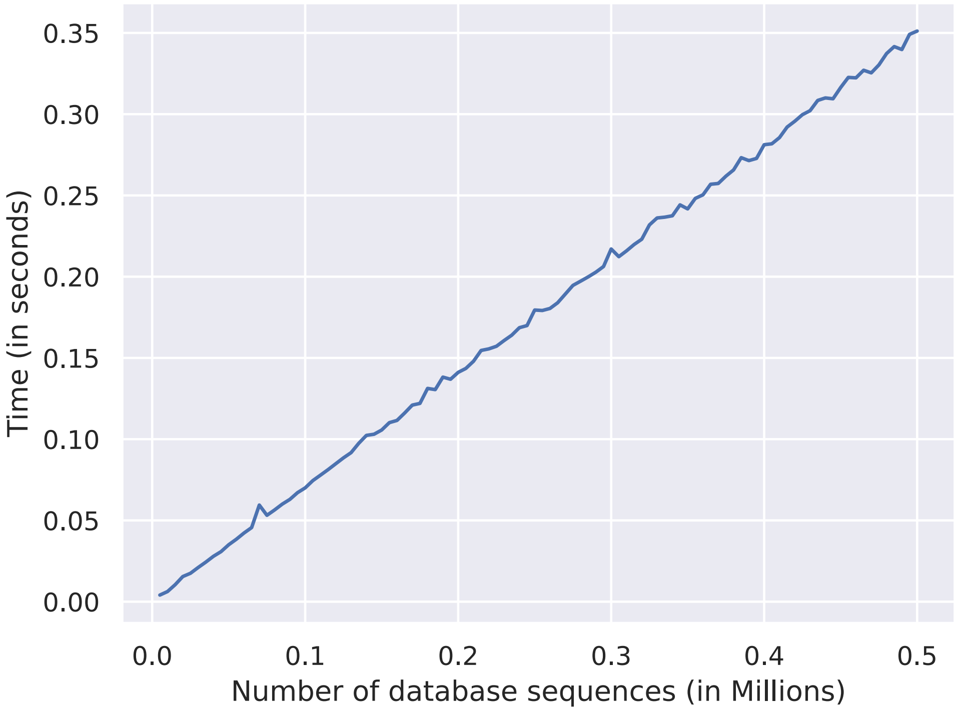

Fig. S2. Empirical runtime requirements of PROST searches. The time complexity of performing a homology search with PROST is linear with the size of the target database. Here we show empirical timing results of PROST with increasing database sizes. Runtime requirement increases in a linear fashion.

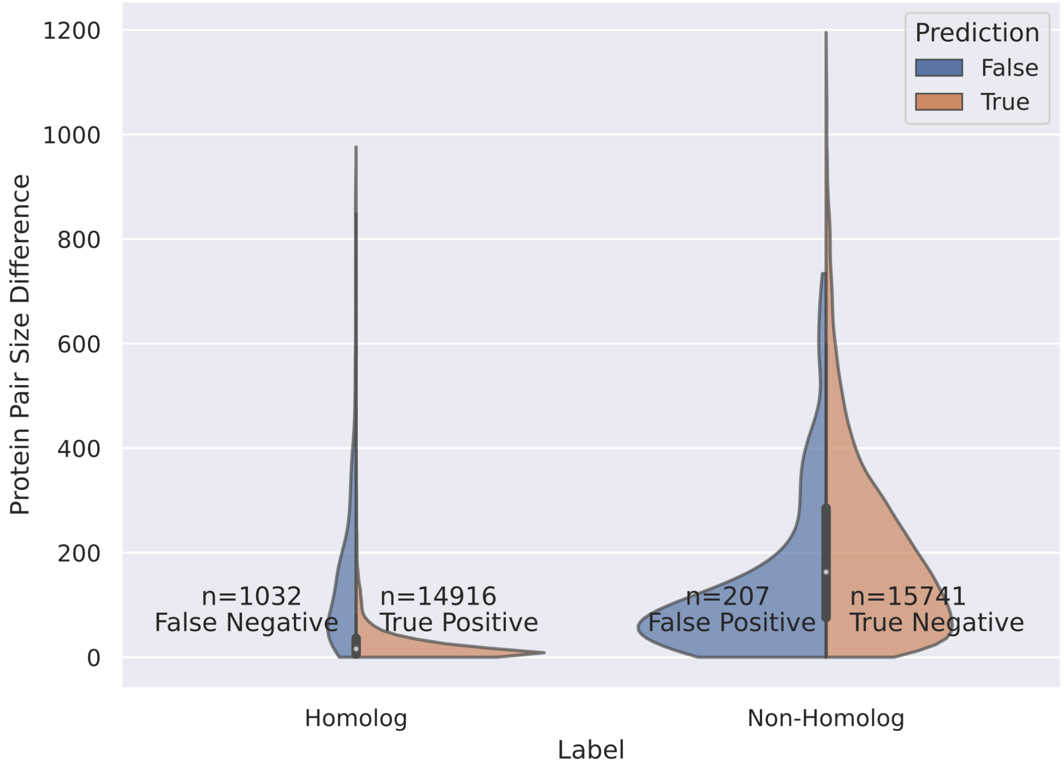

Fig. S3. Prediction outcomes of max50 benchmark protein pairs shown for different protein size differences in the number of residues between two proteins being compared. For example, if a pair of proteins have lengths of 120 and 230 residues are compared, then the difference in length will be 110. Most protein pairs in the benchmarks have similar lengths. This figure shows the distribution of predicted results based on the homology label when a threshold of 6828.5 is used. This threshold is based on the benchmarking dataset found by the maximized F_1 score.

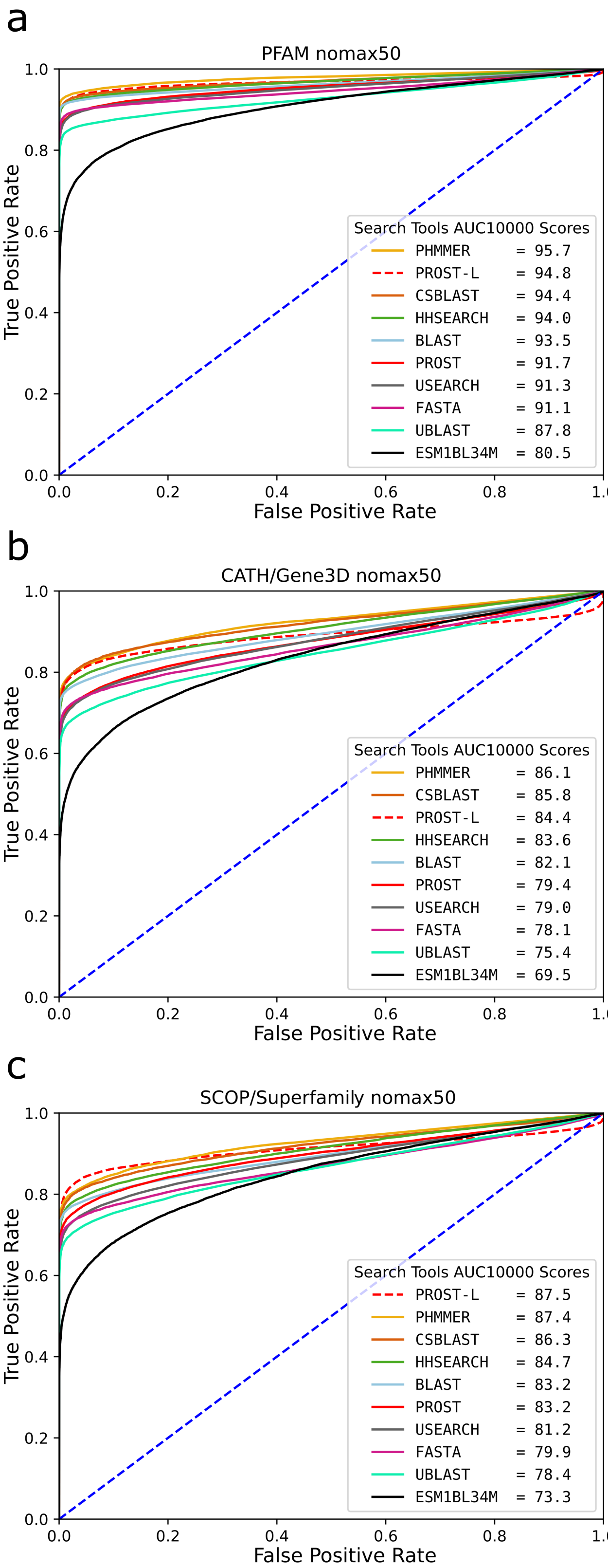

Fig. S4. ROC plots for nomax50 benchmarking dataset. This dataset does not have any length constraint on undefined regions of proteins, and in order for a pair of proteins to be acceptable homologs, they only need to have the same defined domains in consecutive order. But in between defined domains, they may have an unlimited length of undefined amino acid sequences. Due to this, this dataset is similar to a local homology test since the undefined regions may be greater in length than the defined regions. The plots show the overall performance of tested methods as true positive and false positive rates. We ranked each curve based on their performance on the first 10,000 false positives measured by the AUC10000 metric. We used 10,000 false positives because this dataset contains a total of 180,566 pairs. ROC plots for each database (PFAM (a), Gene3d (b), and Superfamily (c)) are shown separately. PROST has a mediocre performance signifying its global alignment nature. However, PROST-L has a performance close to the state of the art. PHHMER performs the best in this benchmark.

Table S1. Effect of different distance metrics on PFAM dataset

Method	PFAM AUC
L1	97.5
L2	97.5
Inner Product	97.3
DTW (3)	97.6
Hausdorff (12)	93.7
Frobenius Norm	97.5

Table S2. Comparison of Homolog Detection Methods in the max50 Dataset

Method	PFAM		Gene3D		SUPERFAMILY	
	AUC	AUC1000	AUC	AUC1000	AUC	AUC1000
PROST-L	99.3	98.2	98.5	95.2	98.7	96.5
**PROST**	**99.0**	**97.2**	**98.9**	**95.7**	**98.5**	**95.5**
CSBLAST (13)	97.0	92.4	96.3	91.0	96.1	90.0
PHMMER (14)	96.4	92.3	96.2	90.4	95.9	90.4
HHSEARCH (15)	96.7	91.7	96.3	90.1	95.1	87.7
NCBI-BLAST (7)	95.9	90.9	94.4	87.8	93.7	85.7
USEARCH (16)	95.2	89.6	94.0	86.2	93.8	85.3
ESM1bL34M (8)	96.0	89.8	91.9	80.1	92.0	81.1
FASTA (17)	94.6	88.8	93.2	85.2	91.9	83.4
UBLAST (16)	93.2	85.6	91.0	81.6	84.2	78.5

Table S3. Comparison of Homolog Detection Methods in the nomax50 Dataset

Method	PFAM		Gene3D		SUPERFAMILY	
	AUC	AUC1000	AUC	AUC1000	AUC	AUC1000
PHMMER (14)	97.8	95.7	92.3	86.1	92.6	87.4
CSBLAST (13)	97.0	94.4	91.9	85.8	91.8	86.3
PROST-L	96.7	94.8	88.9	84.4	91.1	87.5
HHSEARCH (15)	96.8	94.0	90.7	83.6	91.0	84.7
NCBI-BLAST (7)	96.2	93.5	89.4	82.1	89.5	83.2
PROST	95.4	91.7	87.6	79.4	89.7	83.2
USEARCH (16)	95.2	91.3	87.8	79.0	88.6	81.2
FASTA (17)	94.6	91.1	86.5	78.1	87.1	79.9
UBLAST (16)	92.8	87.8	84.9	75.4	86.6	78.4
ESM1bL34M (8)	90.1	80.5	83.6	69.5	84.9	73.3

SI Dataset S1 (ZyncFingerAnalysis.xlsx)

PROST and BLAST search results on Human Zinc finger protein 268 (Q14587) showcases the global alignment nature of the PROST.

References

1. RD Finn, et al., Pfam: the protein families database. *Nucleic Acids Res* **42**, D222–D230 (2013).
2. GV Saripella, ELL Sonnhammer, K Forslund, Benchmarking the next generation of homology inference tools. *Bioinformatics* **32**, 2636–2641 (2016).
3. S Salvador, P Chan, Toward accurate dynamic time warping in linear time and space. *Intell Data Anal* **11**, 561–580 (2007).
4. B Iglewicz, D Hoaglin, *Volume 16: how to detect and handle outliers.* (ASQC Quality Press Milwaukee (WI, USA)) Vol. 16, (1993).
5. JJ Goeman, A Solari, Multiple hypothesis testing in genomics. *Stat Med* **33**, 1946–1978 (2014).
6. T UniProt Consortium, The universal protein resource (uniprot) 2009. *Nucleic Acids Res* **37**, D169–D174 (2009).
7. S Altschul, Basic local alignment search tool. *J Mol Biol* **215**, 403–410 (1990).
8. A Rives, et al., Biological structure and function emerge from scaling unsupervised learning to 250 million protein sequences. *Proc Natl Acad Sci* **118**, e2016239118 (2021).
9. J Jumper, et al., Highly accurate protein structure prediction with alphafold. *Nature* **596**, 583–589 (2021).
10. Z Li, L Jaroszewski, M Iyer, M Sedova, A Godzik, Fatcat 2.0: towards a better understanding of the structural diversity of proteins. *Nucleic Acids Res* **48**, W60–W64 (2020).
11. K Jia, RL Jernigan, New amino acid substitution matrix brings sequence alignments into agreement with structure matches. *Proteins: Struct , Funct , Bioinf* **89**, 671–682 (2021).
12. AA Taha, A Hanbury, An efficient algorithm for calculating the exact hausdorff distance. *IEEE PAMI* **37**, 2153–2163 (2015).
13. A Biegert, J Soding, Sequence context-specific profiles for homology searching. *Proc Natl Acad Sci* **106**, 3770–3775 (2009).
14. RD Finn, J Clements, SR Eddy, Hmmer web server: interactive sequence similarity searching. *Nucleic Acids Res* **39**, W29–W37 (2011).
15. J Soding, Protein homology detection by hmm-hmm comparison. *Bioinformatics* **21**, 951–960 (2004).
16. RC Edgar, Search and clustering orders of magnitude faster than blast. *Bioinformatics* **26**, 2460–2461 (2010).
17. WR Pearson, Rapid and sensitive sequence comparison with fastp and fasta. *Methods Enzym.* **183**, 63–98 (1990).
